# Early Puberty in Greece

**Published:** 1845-01

**Authors:** 


*Early Puberty in Greece.*—Mr. Strong, the Bavarian consul at Athens, in a recent work, (Greece as a Kingdom, 8vo., London,) says: "Nature is so extremely precocious in Greece, that females attain the age of puberty at 10 or 11 years, and men at 15 or 16. Young lads of 16 and 17 are frequently met with in the villages already married and with families. I am acquainted with a lady of one of the first Athenian families, who, though only 25 years of age, has already had sixteen children, (eight of them twins,) of whom seven are still alive. It may scarcely appear credible in England, but there is now at Athens a venerable grandmamma in the person of a lady not yet 24 years old. She was married when 11 years of age, and had a daughter in the course of a year. That daughter married, also, when scarcely 11, and has just become a mother."—*Boston Med. and Surg. Jour.*

